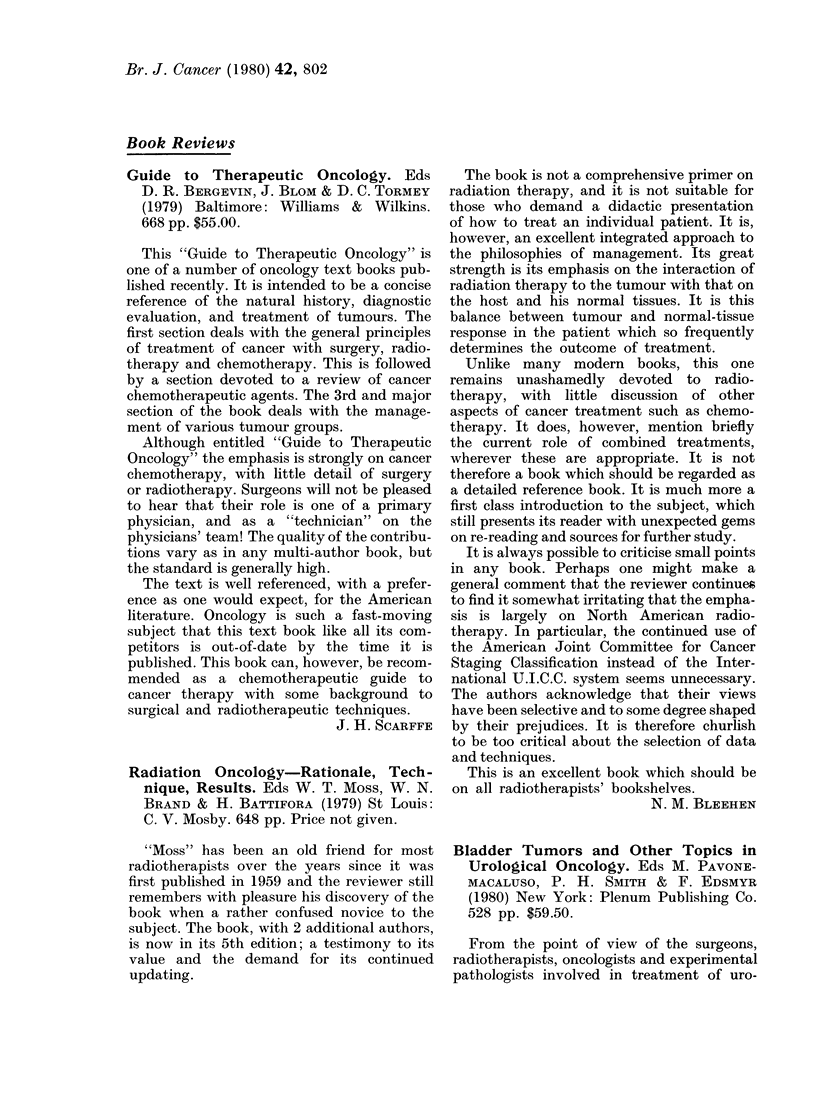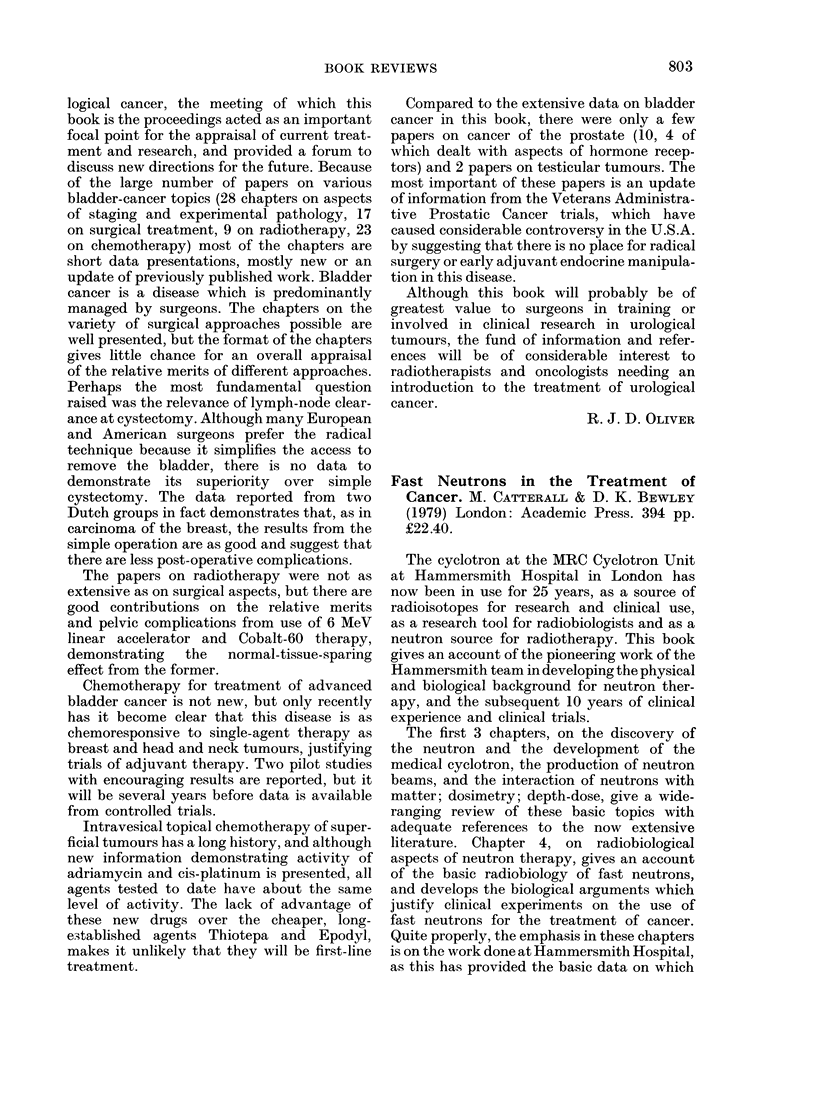# Bladder Tumors and Other Topics in Urological Oncology

**Published:** 1980-11

**Authors:** R. J. D. Oliver


**Bladder Tumors and Other Topics in Urological Oncology.** Eds M. PAVONE-MACALUSO, P. H. SMITH & F. EDSMYR (1980) New York: Plenum Publishing Co. 528 pp. $59.50.

From the point of view of the surgeons, radiotherapists, oncologists and experimental pathologists involved in treatment of urological cancer, the meeting of which this book is the proceedings acted as an important focal point for the appraisal of current treatment and research, and provided a forum to discuss new directions for the future. Because of the large number of papers on various bladder-cancer topics (28 chapters on aspects of staging and experimental pathology, 17 on surgical treatment, 9 on radiotherapy, 23 on chemotherapy) most of the chapters are short data presentations, mostly new or an update of previously published work. Bladder cancer is a disease which is predominantly managed by surgeons. The chapters on the variety of surgical approaches possible are well presented, but the format of the chapters gives little chance for an overall appraisal of the relative merits of different approaches. Perhaps the most fundamental question raised was the relevance of lymph-node clearance at cystectomy. Although many European and American surgeons prefer the radical technique because it simplifies the access to remove the bladder, there is no data to demonstrate its superiority over simple cystectomy. The data reported from two Dutch groups in fact demonstrates that, as in carcinoma of the breast, the results from the simple operation are as good and suggest that there are less post-operative complications.

The papers on radiotherapy were not as extensive as on surgical aspects, but there are good contributions on the relative merits and pelvic complications from use of 6 MeV linear accelerator and Cobalt-60 therapy, demonstrating the normal-tissue-sparing effect from the former.

Chemotherapy for treatment of advanced bladder cancer is not new, but only recently has it become clear that this disease is as chemoresponsive to single-agent therapy as breast and head and neck tumours, justifying trials of adjuvant therapy. Two pilot studies with encouraging results are reported, but it will be several years before data is available from controlled trials.

Intravesical topical chemotherapy of superficial tumours has a long history, and although new information demonstrating activity of adriamycin and cis-platinum is presented, all agents tested to date have about the same level of activity. The lack of advantage of these new drugs over the cheaper, long-established agents Thiotepa and Epodyl, makes it unlikely that they will be first-line treatment.

Compared to the extensive data on bladder cancer in this book, there were only a few papers on cancer of the prostate (10, 4 of which dealt with aspects of hormone receptors) and 2 papers on testicular tumours. The most important of these papers is an update of information from the Veterans Administrative Prostatic Cancer trials, which have caused considerable controversy in the U.S.A. by suggesting that there is no place for radical surgery or early adjuvant endocrine manipulation in this disease.

Although this book will probably be of greatest value to surgeons in training or involved in clinical research in urological tumours, the fund of information and references will be of considerable interest to radiotherapists and oncologists needing an introduction to the treatment of urological cancer.

R. J. D. Oliver